# A Clinical Evaluation of Circulating MiR-106a and Raf-1 as Breast Cancer Diagnostic and Prognostic Markers

**DOI:** 10.31557/APJCP.2021.22.11.3513

**Published:** 2021-11

**Authors:** Elham Ahmed Mohmmed, Wafaa Ghoneim Shousha, Abeer Salah EL-Saiid, Shimaa Shawki Ramadan

**Affiliations:** 1 *Department of Central Labs, National Nutrition Institute, Cairo, Egypt. *; 2 *Department of Biochemistry, Faculty of Science, Helwan University, Cairo, Egypt. *; 3 *Department of Clinical and Chemical Pathology, National Cancer Institute, Cairo University, Cairo, Egypt. *

**Keywords:** Breast cancer, lymph node, metastasis, MicroRNA-106a, WBCs, platelets and RAF-1

## Abstract

**Objective:**

MicroRNAs (MiRNAs) regulate mammalian cell growth, differentiation, and apoptosis by altering the expression of other genes and serve multiple roles in tumorigenesis and progression. Proto-oncogene serine/threonine-protein kinase (RAF-1) functions as a part of the MAPK/ERK signal transduction pathway. The present study aim was to prospectively evaluate MicroRNA 106a (MiR-106a) and RAF-1 as a diagnostic and prognostic factor in early prediction of breast cancer (BC), recurrence and early detection of distant metastasis as well as to analyses the statistical correlation between MiR-106a and RAF-1 levels and clinical-pathological parameters including tumor size, lymph node, histological type and grading.

**Methods:**

Sera and plasma of 30 normal women and 50 women with breast carcinoma were assayed for MiR-106a by RT-qPCR as well as levels of Hb, WBCs and platelets count and RAF-1 by solid phase enzyme-linked immunosorbent assay (ELISA).

**Results:**

The patients’ characteristics, they were classified according to grade into 8% grade I, 66% grade II, 22% grade III and 4% grade IV. The stages were classified according to the TNM system as stage II was the highest percentage 66%, while the lowest percentage was 10% for stage I and 24% for stage III. Also, Hb% and RAF-1 levels were significantly decreased in breast cancer patients as compared with healthy control. On the other hand, MiRNA-106a gene expression was non-significantly increased in positive lymph node metastasis patients (FC=3.66) when compared to patients with negative lymph node metastasis (FC=3.51). In addition, MiR-106a was significantly up-regulated in breast cancer patients with a fold of change 3.63 when compared to control samples.

**Conclusion:**

Expression of MiR-106a gene can be used as a diagnostic and prognostic noninvasive biomarker which can stimulates breast cancer cell invasion and proliferation through downregulation of Raf-1 levels.

## Introduction

Breast cancer is the most common cancer among women worldwide, and 70–80 percent of patients with early-stage, non-metastatic disease can be cured (Harbeck et al., 2019). Breast cancer is a personal tragedy for those who are diagnosed. When detected early, it is a highly curable illness, but when detected late, it’s almost always fatal. Early diagnosis and access to the best treatment may mean life or death (El Saghir and Anderson, 2012; Milosevic et al., 2018). According to the GLOBOCAN 2020 evaluations of cancer occurrence and mortality formed by the International Agency for Research on Cancer, breast cancer in females has exceeded lung cancer as the most cancer commonly diagnosed, with 2.3 million new estimated cases (11.7%), followed by lung (11.4%) (Sung et al., 2021b). Death rate for female breast cancer was noticeably elevated in transitioning versus transitioned countries (15.0 vs 12.8 per 100,000)(Sung et al., 2021a). Only 25–30% of women with metastatic breast cancer have a 5-year survival rate (Mondal and Meeran, 2020) and 80% of women with early breast cancer now surviving 10 years after diagnosis (Chopra and Davies, 2020).

MiRNAs are small, non-coding and highly abundant molecules that have been shown to play critical regulatory roles in a wide range of biological and pathological processes (Lee et al., 1993; Jackson and Standart, 2007; Pawlick et al., 2021). MiRNAs may regulate cellular gene expression at the transcriptional or post-transcriptional level; by suppressing translation of protein coding genes, or cleaving target MiRNAs to induce their degradation, through imperfect pairing with target mRNAs of protein coding genes (Bartel, 2004; Nachtigall and Bovolenta, 2022). Circulating MiRNAs are becoming more widely used as effective biomarkers in a variety of diseases, including breast cancer (Jang et al., 2021).

Several studies have explored the differential expression of circulating MiRNAs in BC patients, but very few results are reproducible among laboratories due to population

diversity and lack of standardized protocols about applying MiRNAs in recent clinical practice (Witwer, 2015; Condrat et al., 2020). Aberrant expression of certain MiRNAs has been observed in an array of human cancer types, and MiRNAs are thought to serve important roles in tumorigenesis. Also, a few MiRNAs have been identified as oncogenes or tumor suppressor genes in BC (Hayashita et al., 2005; Ventura et al., 2008; Olive et al., 2010; Chan et al., 2013; Battistella et al., 2015). MiR-106a is expressed in stool samples and in cancer cells of patients with BC with varying degrees (Jung et al., 2012; Schwarzenbach et al., 2014; Kleivi Sahlberg et al., 2015; Mishra et al., 2015; Hamam et al., 2017). 

The receptor tyrosine kinase effector, Raf, named for Rapidly Accelerated Fibrosarcoma, was discovered over two decades ago by two groups independently as a retroviral oncogene, v-Raf or v-MIL, possessing a serine/threonine kinase activity (Baccarini, 2005; Simanshu et al., 2017). Raf-1 Kinase Inhibitor Protein (RKIP) is one of these promising metastasis suppressors, which has various physiologic functions (Hagan et al., 2005; Muslin, 2005; Pottier et al., 2020) . The collective evidence indicates that RKIP regulates the activity and mediates the crosstalk between several important cellular signaling pathways, including the Raf/MAP/extracellular signal regulated kinase (MAPK)/ERK pathway (Galabova-Kovacs et al., 2006; Pottier et al., 2020), nuclear factor-κB (NF-κB) pathway , and G-protein pathway (Kroslak et al., 2001; Shvartsur et al., 2017; El-Fadl et al., 2021).

Breast cancer growth and progression are promoted by aberrant activation of the Raf/MEK/MAPK pathway. Overexpression of the HER-2/Neu tyrosine kinase receptor causes dysregulation of Raf/MEK/MAPK oncogenic signalling, which leads to chemoendocrine resistance, the development of distant metastases, and ultimately a poor prognosis in breast cancer patients (Leontovich et al., 2012). The present study aimed to evaluate the correlation between MiRNA-106a gene expression and levels of Hb%, WBCs and platelets count as well as tumor marker proteins RAF-1 in a trial to check their early diagnostic and prognostic value which may help to understand their roles in breast cancer.

## Materials and Methods

*Patients and Methods*

In this prospective non-randomized study, we enrolled 80 females from the National Cancer Institute, Cairo University. The average age was 26-64 years. Informed consent was obtained from all patients and controls. Group I was composed of 50 breast cancer patients. Group II included 30 healthy controls. On the other hand, the study protocol was approved by the local ethics committee, and informed written consent was obtained from the parents of the patients and volunteers before entering the study.

Inclusion criteria for both groups were females age (26-64 years) when newly diagnosed according to AJCC staging system (7th Edition) classification: Information concerning age, diagnosis and clinical pathology such as tumor size (T), lymph node status (N), grade and hormonal status, for each patient were collected before any treatment through clinical charts (Table 1). 

Exclusion criteria included presence of autoimmune disease, acute kidney injury or with unsatisfactory vascular access or any other known condition that would alter growth hormones levels. Moreover, none of our patients had received antibiotics, anti-inflammatory or corticosteroid medications during the study period. 

Clinical assessments included complete history taking, past medical and disease history for confirming the appropriateness of the patients to the inclusion criteria. 

*Blood sampling and Biochemical analyses*

*Sample collection*

The blood samples were collected from Egyptian breast cancer patients and healthy control women during the period 2017 and 2018. Blood samples were gathered in heparinized and non-heparinized tubes. The heparinized tube sample was divided into 2 parts. the first one analyzed for prognostic significance of peripheral blood. The different parameters like Hb concentration, WBCs and blood platelet count were included in this study. The second part was centrifugated and plasma separated for MiRNA-106a using RT-qPCR. The non-heparinized tubes were allowed to Hemoglobin clot at room temperature for 10-20 min, and sera were then separated by centrifugation (2,000-3,000 rpm, 20 min, 25°C) was stored at -20°C for estimation of serum RAF-1 levels was determined by Enzyme Immunoassay Kit based on the principle of a solid phase enzyme-linked immunosorbent assay (ELISA), purchased from SinoGeneClon Biotech Co., Ltd, Hangzhou, China. 

*MiRNA-106a analysis using RT-qPCR*

Total RNA was obtained by using QIAamp RNA blood Mini Kit Cat. No. 52304 (Qiagen, Düsseldorf, Germany) following the manufacturer’s commands. Total RNA was transcribed reversely using (Thermo Fisher) (200 U/ µL), Cat. No. EP0441 following the manufacturer’s instructions and complementary DNA (cDNA) synthesis was done in the thermal cycler. Real-time PCR was done using Quantitect SYBR green PCR kit Cat. No. 204141 according to the manufacturer’s instructions (Table 2). RNA specific primers for MiR-106a and housekeeping gene (Human GAPDH) are shown in (Table 3). The results of the SYBR green RT-PCR were analyzed as follows: Gene expression levels were calculated using the Stratagene MX3005P instrument, which provided amplification curves and Ct values.

*Statistical analysis*

All the grouped data were statistically evaluated with SPSS^©^ Statistics version 27 (IBM^©^ Corp., Armonk, NY, USA). c omparison between two groups was performed using an independent sample t-test. While comparison between three groups was done using one way ANOVA test. P values of less than 0.05 were considered to indicate statistical significance. All the results were expressed as mean ± SEM. MiR-106a expression and fold change (FC) were computed using the standard formula (Livak and Schmittgen, 2001). The correlations between numerical variables were estimated by Pearson’s correlation coefficient (r) determination.

## Results

This analysis focused on 50 patients with breast carcinoma. The mean age was 49.7±2.08 as illustrated in Table 1. The patients’ characteristics, they were classified according to grade into 8% grade I, 66% grade II, 22% grade III and 4% grade IV. The stages were classified according to the TNM system as stage II was the highest percentage 66%, While the lowest percentage was 10% for stage I and 24% for stage III. The classification according to tumor size was > 5 cm in 10% of patients, < 2 cm in 16% and, between 2-5 cm in 74%.

Hb level was noted in breast cancer patients. Hb count was found to be in the range of 9-14 gm/dl. The mean of Hb level was significantly decreased (9.58±0.19) as compared to the mean value of Hb level in healthy control (12.81±0.26) (P=0.000). The level of Hb falls, and this may cause the risk of anemia in the breast cancer patients (Table 4). The mean of WBCs count level was significantly increased (6.64±0.11) x 10^3^ /mm^3^ as compared to the mean value of WBCs in healthy control (5.19±0.32) x 10^3^ /mm^3^ (P=0.000). The platelet count of peripheral blood has an important diagnostic significance. The majority of patients were found with (392.90±8.05) x 10^3^ /mm^3^ as compared to the mean value of Platelet count in healthy control (291.78±0.20) x 10^3^ /mm^3^ (P=0.000) (Table 4).

Table 4 showed the serum RAF-1 levels of breast cancer patients and health control females. RAF-1 levels were significantly decreased in each patient (61.50±1.10 pg/ml) as compared to mean value of RAF-1 in healthy control (68.51±2.81 pg/ml) (P=0.011). In other hand Raf-1 serum level was significantly increased in patients have estrogen and progesterone receptors as compared to patients with no receptors (p=.016; 0.174 respectively) as in Table 4. 

*Identification of Deregulated MiRNA by the ΔΔ Ct Method*

Input data from the Gene Globe data analysis tool were computed using the standard delta–delta Ct method (ΔΔ Ct) with GAPDH Ct as references. Fold change was calculated, and the homogeneity of data was visualized. Up- or downregulation of MiRNA was confirmed in 100% of analyzed samples when compared to the Gene Globe final experiment (Table 5). The ΔΔ Ct method identified MiR-106a delta Ct was significantly decreased in breast cancer patients (-1.2050±0.11511) when compared to control samples (0.53±0.41) (P=0.000) as in Table 4. Also, it was observed that, MiR-106a gene was highly expressed in breast cancer patients (FC=3.63) as in table 3 which showed that the relative expression of MiR-106a gene in BC patients was significantly increased compared to healthy control females. 

**Figure 1:**
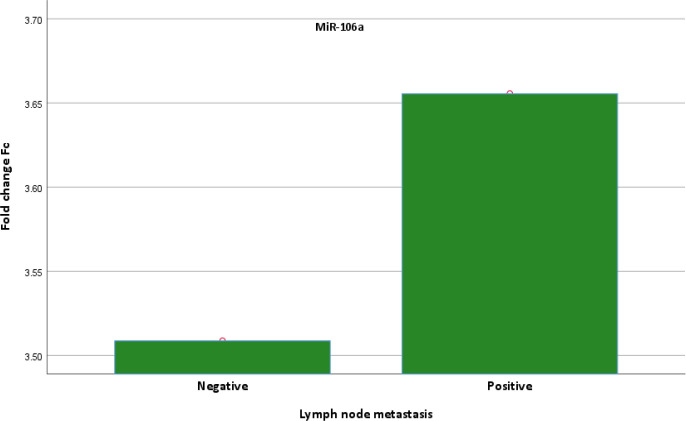
Graph of Fold Change of MiR-106a for Negative and Positive Lymph Node Metastasis

Also, data in Table 6 illustrated that, the expression of MiR-106a gene was increased in grade IV (FC=3.83) compared to grade III (FC=3.34) but the increase was not statistically significant. In addition, the expression of MiR-106a gene was decreased in stage III (FC=3.32) compared to grade II (FC=3.73) but the decrease wasn’t statistically significant. The data also clarified that, MiR-106a gene expression in positive lymph node metastasis patients (FC=3.66) was higher than that of the negative lymph node metastasis patients (FC=3.52) but the increase was regarded as statistically non-significant as illustrated in Figure 1.

Table 7 showed the correlation between MiR-106a FC and Hb%, WBC, Platelets and RAF-1 in the breast cancer patients. The present results indicated that a non-significant negative correlation was observed between MiR-106a and Hb% (r = - 0.059, p <0.758).

In addition, our results showed that a non-significant negative correlation was observed between MiR-106a and WBCs (r = - 0.057, p <0.763). on the other hand, a non-significant positive correlation was observed between MiR-106a and Platelets (r = + 0.010, p <0.959). 

**Figure 2:**
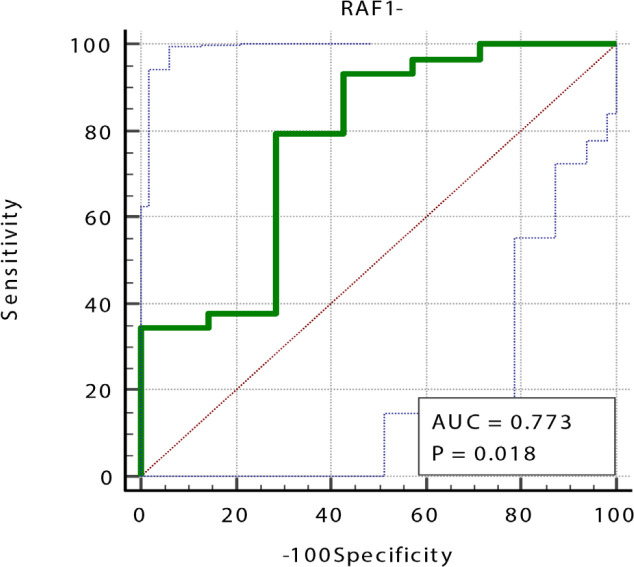
ROC Curve of RAF-1 in BC Patients Compared to Healthy Control

**Figure 3:**
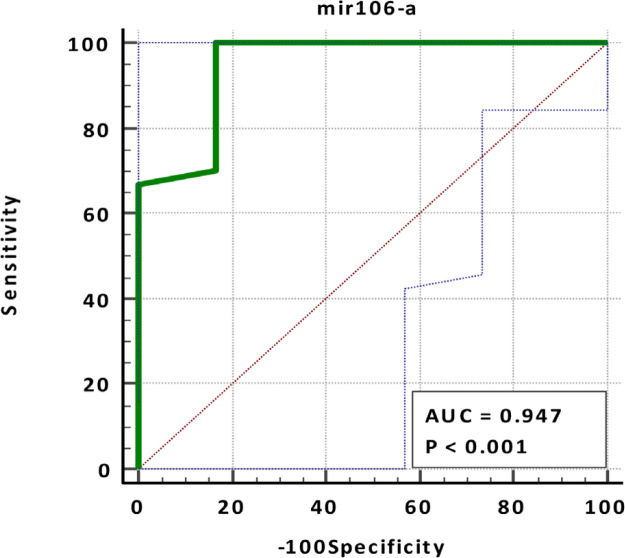
ROC Curve of MiR-106a in BC Patients Compared to Healthy Control

 Also, a non-significant negative correlation between MiR-106a FC and RAF-1 was noted (r = -0.191, p <0.322). Table 8 and Figures 2 and 3, showed that MiR-106a FC showed a very high diagnostic value (AUC = 0.947, specificity = 83.33%, sensitivity = 100%, p-value =0.0001) in opposite to RAF-1 which showed a very low diagnostic value (AUC =0.773, specificity = 71.43%, sensitivity = 79.31%, p-value =0.081).

**Table 1:** Main Clinical-Pathological Characteristics of 50 Breast Cancer Patients

Parameters N %	N	%
Age mean 49.7±2.08.	50	100
Tumor size		
T1 < 2	8	16
T2 2–5	37	74
T3 > 5	5	10
Auxiliary lymph node		
Positive	42	84
Negative	8	16
Pathological grade		
Grade I	4	8
Grade II	33	66
Grade III	11	22
Grade IV	2	4
Clinical stage		
Stage 1	5	10
Stage 2	33	66
Stage 3	12	24
Pathological type		
IDC	40	80
ILC	5	10
ICC	4	8
Estrogen receptor		
Positive	20	40
Negative	30	60
Progesterone receptor		
Positive	17	34
Negative	33	66
HER-2		
Positive	13	26
Negative	37	74
Family History		
Positive	12	24
Negative	38	76

**Table 2:** SYBR Green Real-Time PCR Cycling Conditions

Gene	MiR-106a		GAPDH	
	Time	Temp (°C)	Time	Temp (°C)
Reverse transcription	30 min	50˚C	30 min	50˚C
Primary denaturation	5 min.	94˚C	5 min.	94˚C
Amplifcation (40 cycles)				
Secondary denaturation	15 sec	94 ˚C	15 sec	94 ˚C
Annealing (optics on)	30 sec.	58 ˚C	30 sec.	58 ˚C
Extension	30 sec.	72 ˚C	30 sec.	72 ˚C
Dissociation curve (1 cycle)				
Secondary denaturation	1 min.	94 ˚C	1 min.	94 ˚C
Annealing	1 min.	58 ˚C	1 min.	58 ˚C
Final denaturation	1 min.	94 ˚C	1 min.	94 ˚C

**Table 3:** RNA Specifc Primers for MiR-106a Gene and Housekeeping Gene (Human GAPDH)

Gene	Primer sequence (5'-3')	Reference
Human GAPDH	CTCTGATTTGGTCGTATTGGG	(Li et al., 2014)
	TGGAAGATGGTGATGGGATT	
MiR-106a	ATCCAGTGCGTGTCGTG	
	TGCTAAAAGTGCTTACAGTG	
	GTG CAG GGT CCG AGG T	

**Table 4:** The Levels of HB, Platelets, WBCs, RAF-1 and MiR-106a delta Ct of Breast Cancer Patients and Healthy Control

Parameter	Patients	Health control	P-value
	Mean ±SE	Mean ±SE	
HB (gm/dl)	9.58±0.19	12.81±0.26	0.000**
Platelets (x10^3^ /mm^3^)	392.90±8.05	291.78±0.20	0.000**
WBCs (x10^3^ /mm^3^)	6.64±0.11	5.19±0.32	0.000**
RAF-1(pg/ml)	61.50±1.10	68.51±2.81	0.011*`
MiR-106a delta Ct	-1.2050±0.11511	0.53±0.41	0.000**

Data were expressed as mean ± standard Error. *, is significant; **, is highly significant.

**Table 5:** Fold Change Data Analysis of Tested Circulating MiR-106a in the Serum of Cancer Patients

	GAPDH Ct	MiR-106a Ct	Δ Ct	ΔΔ Ct	FC
Compared to the healthy control group					
Control	27.29	27.82	0.53	0	1.00
Patients	25.78	24.57	-1.20	-1.73	3.63
Pathological grade					
Grade I	23.30	22.32	-0.99	-1.51	2.85
Grade II	26.04	24.79	-1.24	-1.77	3.80
Grade III	26.03	24.90	-1.13	-1.65	3.34
Grade IV	23.86	22.45	-1.41	-1.94	3.83
Clinical Stage					
Stage I	24.76	23.52	-1.25	-1.77	3.73
Stage II	25.93	24.69	-1.24	-1.77	3.73
Stage III	25.77	24.70	-1.08	-1.60	3.32
Pathological type					
IDC	25.89	24.62	-1.27	-1.80	3.80
ILC	25.87	24.67	-1.21	-1.73	3.47
ICC	23.30	22.32	0.99	-1.51	2.85
Auxiliary lymph node					
Positive	25.98	24.78	1.20	-1.73	3.66
Negative	24.75	23.59	-1.20	-1.73	3.51
Tumor size					
T1 < 2	25.38	24.12	-1.26	-1.79	3.80
T2 2–5	25.92	24.76	-1.16	-1.69	3.54
T3 > 5	25.40	23.98	-1.43	-1.95	4.05
Estrogen receptor					
Negative	25.82	24.50	-1.32	-1.84	3.95
Positive	25.72	24.68	-1.04	-1.57	3.16
Progesterone receptor					
Negative	25.67	24.38	-1.30	-1.82	3.87
Positive	25.99	24.96	-1.02	-1.55	3.15
HER-2					
Positive	25.42	24.16	-1.26	-1.78	3.83
Negative	25.90	24.72	-1.19	-1.71	3.56
Family History					
Yes	26.40	24.99	-1.41	-1.94	4.16
No	25.59	24.44	-1.14	-1.67	3.47

**Table 6:** Correlation between MiR-106a and RAF-1 with Clinical-Pathological Data of Breast Cancer Patients

	RAF-1	MiR-106a FC
Pathological grade		
І	58.15±6.97	2.85±0.11
ІІ	61.90±1.29	3.80±0.39
ІІІ	62.07±2.59	3.34 ±0.49
ІV	56.75±0	3.83±0
P-value	0.719	0.817
Clinical Stage		
І	64.94±2.54	3.73±3.32
ІІ	61.32±1.47	3.73±0.36
ІІІ	61.02±1.66	3.32±0.52
P-value	0.705	0.839
Pathological type		
IDC	61.94±1.18	3.81±0.33
ILC	59.91±4.85	3.47 ±0.76
ICC	58.15±6.97	3.47 ±0.11
P-value	0.638	0.683
Auxiliary lymph node		
Positive	61.88±1.15	3.66±0.32
Negative	59.098±3.63	3.51±0.62
P-value	0.392	0.85
Tumor size		
T1 < 2	63.07± 3.14	3.80±0.79
T2 2–5	61.21± 1.36	3.47±0.35
T3 > 5	59.89 ± 2.82	4.06±0.79
P-value	0.781	0.795
Estrogen receptor		
Negative	63.66±1.42	3.95±0.41
Positive	58.44±1.35	3.17±0.32
P-value	0.016*	0.174
Progesterone receptor		
Positive	58.07±1.61	3.15±0.38
Negative	63.30±1.23	3.87±0.37
P-value	0.021*	0.235
HER-2		
Positive	61.49±1.70	3.83±0.70
Negative	61.50±1.40	3.56±0.30
P-value	0.998	0.681
Family History		
Yes	56.36±1.38	4.17±0.73
No	63.13±1.19	3.47±0.30
P-value	0.006**	0.304

Data were expressed as mean ± standard Error. *, is significant **, is highly significant.

**Table 7:** Correlation between MiR-106a FC and Hb%, WBC, Platelets & RAF-1 in the Breast Cancer Patients

		HB	WBC	Platelets	RAF-1
WBC	r	0.107			
	p	0.573			
Platelets	r	0.206	-0.323		
	p	0.274	0.082		
RAF-1	r	0.131	-0.155	-0.044	
	p	0.498	0.423	0.823	
MiR-106a FC	r	-0.059	-0.057	0.010	-0.191
	p	0.758	0.763	0.959	0.322

r, correlation coefficient; P-value, probability value; *, is significant; **, is highly significant.

**Table 8:** The Sensitivity, Specificity, Cut off, and AUC (Area under Curve) for MiR-106a FC and RAF-1 in Breast Cancer Patients

Parameters	AUC	P-value	95% Confidence Interval		Cut off	Sensitivity	Specificity
			Lower Bound	Upper Bound	Value		
MiR-106a Fold change	0.947	<0.0001	0.817	0.994	>1.45	100	83.33
RAF-1	0.773	0.018	0.604	0.896	≤66.08	79.31	71.43

## Discussion

In women, breast cancer is the leading cause of cancer-related death. Breast cancer-related mortality is mainly caused by recurrence of the primary tumor and metastasis to distant body parts (Mondal and Meeran, 2020). Gene expression variations have resulted in the emergence of recent technical advances in cancer diagnosis, allowing for a better understanding of tumor behavior. It has improved not only the prognosis, but also the early detection and treatment of cancer (Latha et al., 2020). BC has been shown to have abnormal MiRNA cluster expression, which can be both pro-tumorigenic and anti-tumorigenic. So, the use of circulating MiRNAs in breast cancer diagnosis is less invasive and efficient than traditional tissue biopsy(Yoshikawa et al., 2018; Swellam et al., 2019). The role of MiR-106a in the development of tumor malignancy is complex and controversial. Several studies have shown that MiR-106a can act as a tumor suppressor or oncogene in various cancer types, depending on the cellular context (Yang et al., 2011; Kim et al., 2012). In this study, we focused on MiR-106a, which is located on chromosome X and has received little attention in BC despite the strong correlation between sex and the incidence of BC well as its correlation with RAF-1 levels (Li et al., 2018). Our study demonstrated that the overexpression of MiR-106a induced proliferation and decreased apoptosis in cells could be by inhibition of RAF-1 gene expression. It’s approved that MiR-106a overexpression stimulates breast cancer cell invasion and proliferation through upregulation of Bcl-2, ABCG2, and P53, and downregulation of Bax and RUNX3. (You et al., 2019). At the same time, studies showed that Proto-oncogene serine/threonine-protein kinase (Raf-1) functions as a part of the MAPK/ERK signal transduction pathway. Once activated, Raf-1 phosphorylates and activates MEK1 and MEK2 protein kinases and then, in turn, phosphorylates and activates the serine/threonine-specific protein kinases ERK1 and ERK2 to control expression of various genes (such as Bcl-2 and P-glycoprotein) in the regulation of cell cycle, cell migration, apoptosis and differentiation (Hoyle et al., 2000; Cekanova et al., 2007). 

In previous assessed breast tumors, corresponding serum from patients showed that MiR-106a was significantly overexpressed in both breast tumors and corresponding serum samples(Li et al., 2018; Zhang et al., 2021). The expression was higher in negative progesterone receptor versus positive patients, and also in negative estrogen receptor versus positive ER patients (Wang et al., 2010). The result of our study showed that, MiR-106a gene was elevated in negative progesterone receptor as compared to positive patient (Fc=3.87 and 3.15, respectively) and MiR-106a gene was raised in negative estrogen receptor as compared to positive patient (Fc=3.95and 3.16, respectively).

Moreover MiR-106a slows monocyte and then macrophage growth (Fontana et al., 2007). That could decrease the primary clearing reactions to altered cells and may increase the prevalence of breast cancer. It is approved that, in the intrinsic pathway for apoptosis initiation, caspase 6 is the direct activator of caspase 8 (Cowling and Downward, 2002). A reduction in caspase 6 and caspase 8 expression induced by elevated MiR-106a would be expected to reduce apoptosis, resulting in an increase in cell number (Vakkala et al., 1999).

In conclusion, the results of the current study demonstrated that MiR-106a functions as an oncogene and increases in BC cancers. The MiR-106a clearly targets genes involved in tumorigenesis, proliferation, invasion, migration, and metastases. So, the MiR-106a gene may serve as a potential genetic noninvasive biomarker in breast cancer patients, through regulating RAF-1 expression. However, in vivo studies of related cytokines and inflammatory mediators are warranted to validate these findings.

## Author Contribution Statement

All the authors contributed equally to this work. 

## Ethical approval

All procedures performed in the study involving human participants were in accordance with the ethical standards of the ethics committee of National Cancer Institute, Cairo University, Egypt (No. 201716064.4). 

## Conflict of interest

The authors declare that they have no conflict of interest.
